# Mycoplasma-pneumonia-induced Stevens–Johnson syndrome in an adult: a case report

**DOI:** 10.1186/s13256-024-04758-y

**Published:** 2025-02-26

**Authors:** Fathima Thesleem Yoosuf, Bassem Al Hariri, Memon Noor Illahi, Muhammad Sharif, Muhammad Yousaf, Mohamed Gaafar Mohamedali, Muayad Kasim Khalid

**Affiliations:** 1 Hamad Medical Corporation (HMC), P.O. Box 3050, Doha, Qatar; 2 Weill Cornell Medicine – Qatar, Ar-Rayyan, Qatar; 3 Qatar University Medicine College, Doha, Qatar

**Keywords:** Stevens–Johnson syndrome, Mycoplasma pneumoniae, Mucositis, Rash, Case report

## Abstract

**Background:**

Stevens–Johnson syndrome epitomizes an acute, exceptionally rare, and capricious immunological phenomenon marked by potentially life-threatening skin reactions, involvement of mucous membranes, and concomitant systemic manifestations. Most cases of Stevens–Johnson syndrome have been attributed to being triggered by drugs, while a minority have implicated infectious agents such as Mycoplasma pneumoniae and Coxsackie virus A6 as their cause. We present a case report on the rare occurrence of Mycoplasma-pneumoniae-induced Stevens–Johnson Syndrome in a 25-year-old Sri Lankan male adult.

**Case presentation:**

A 25-year-old Sri Lankan male adult sought medical attention at our institution, presenting a constellation of symptoms composed of fever with chills, dyspnea, pleuritic chest pain, cough producing reddish sputum, and sore throat, persisting over a 4-day period; 2 days following the onset of the respiratory symptoms, he experienced ocular congestion with purulent discharge and painful oral lesions. He had associated generalized body ache and fatigue.

Stevens-Johnson syndrome is diagnosed by skin biopsy.

**Conclusion:**

Stevens–Johnson syndrome is an acute and debilitating condition that requires prompt and timely management to ensure minimum morbidity of the patient. The similarities and overlap of features between Stevens–Johnson syndrome caused due to infectious and drug-related etiologies pose a diagnostic challenge for the physicians, which needs to be subdued using systematic research and evaluation with subsequent formulation of an evidence-based assessment and management plan to ensure safe and efficacious medical care for the patients.

## Introduction

Mycoplasmas are the smallest self-replicating organisms with the smallest genomes, and even so, the species *M. pneumoniae* emerges as the leading culprit behind atypical pneumonia cases. While the majority of infections present with mild symptoms, the emergence of extrapulmonary complications, particularly those involving mucocutaneous manifestations, has been well documented. *Mycoplasma pneumoniae* is the infectious agent most commonly associated with Stevens–Johnson syndrome (SJS). Existing literature on this topic has been predominantly described in children and young people. Hence, our aim here is to report a peculiar case in a 25-year-old Sri Lankan male patient who grappled with this debilitating condition.

### Case presentation

A 25-year-old Sri Lankan male patient sought medical attention at our institution, presenting with a constellation of symptoms composed of fever with chills, dyspnea, pleuritic chest pain, cough producing reddish sputum, and sore throat, persisting over a 4-day period; 2 days following the onset of the respiratory symptoms, he experienced ocular congestion with purulent discharge and painful oral lesions. He had associated generalized body ache and fatigue. He denied having any nasal congestion, rhinorrhea, or genital lesions, with no recent travel history, sick contacts, or ongoing medication regimens. He claimed to be a non-smoker and disclosed that his occupation was that of a school cleaner. Crucially, he reported no history of drug allergies.

**Fig. 1:**
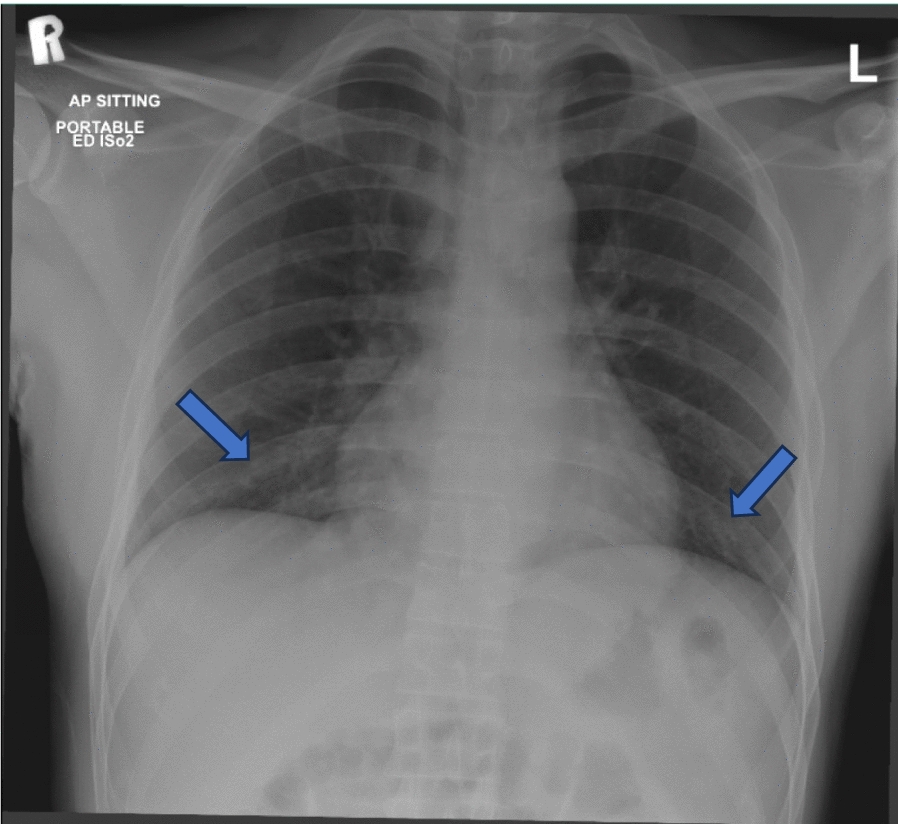
Chest X ray showing subtle bilateral infiltrates (blue arrows)

His vital signs revealed a low-grade fever of 38.1 ℃, accompanied by a rapid heart rate of 122 beats per minute, elevated respiratory rate of 26 breaths per minute, blood pressure within normal limits (115/71 mmHg), and 100% oxygen saturation while on oxygen supplementation via a non-rebreather mask at a flow rate of 10 L per minute. Despite his distress, the patient remained alert and oriented. Physical examination revealed hyperemic eyes with increased lacrimation, in conjunction with dry and cracked lips with black-colored crusting over them. On oral examination, he had ulcers on the buccal mucosa and hard palate with uvula-sparing and serosanguineous secretions in his mouth. His throat appeared pink. Pulmonary auscultation unveiled crepitation in the right intrascapular region with bilateral equal air entry. Cardiac and abdominal examinations were unremarkable, and skin examination revealed warmth without erythema or edema, and no signs of genital lesions. Laboratory investigation was significant for an elevated C-reactive protein (CRP) level of 223 (Table 1) and his chest X-ray (Fig. 1) demonstrated subtle bilateral infiltrates. Subsequently, the viral respiratory panel came back positive for Mycoplasma pneumoniae. He was started on ceftriaxone with azithromycin and diagnosed with sepsis secondary to Mycoplasma pneumoniae with bilateral conjunctivitis.

**Table 1:** Blood parameters

Parameter	On admission (day 1)	Before initiating steroid therapy (day 3)	After 1 week of steroid therapy (day 10)	On discharge (day 13)
WBC	3.6	4.3	12.6	14
ESR	24	–	–	–
CRP	223	106	–	–
LDH	301	–	–	–
CK	729	413	–	–

The next day, the patient was found to have developed papules with central darkening scattered across his upper chest and back, alongside blisters and bullae encompassing less than 10% of his body surface area, primarily localized to his right flank. He also reported having odynophagia while both eating and drinking. Nikolsky’s sign was negative with absence of urogenital involvement. A dermatological assessment was performed, and he was diagnosed as a case of Mycoplasma-induced Stevens–Johnson Syndrome. He was started on triple therapy with intravenous immunoglobulin 1 g/kg for 3 days, intravenous dexamethasone 0.1 mg/kg and intravenous cyclosporine 3 mg/kg in conjunction to the antibiotics. He was also maintained on intravenous fluids to ensure adequate hydration. Dressing with emollients were applied to maintain barrier protection. Further investigation including anti-skin antibody testing, wound culture, and punch biopsy from blister on the right flank was carried out (Table 2). He was placed under protective isolation on a clear fluid diet.

**Table 2:** Diagnostic tests

Diagnostic test	Results
ANCA	Negative
ANA	Negative
Anti-skin Ab IgG	Negative
Skin Ab report	Negative for pemphigoid and pemphigus
Viral screen PCR	Positive for Mycoplasma pneumoniae
Viral serology	Negative for: HSV I HSV II EBV
Hepatitis screen	Negative
HIV serology	Non-reactive
Sputum culture	Negative
Blood culture	Negative
Urine culture	Negative
Wound culture	Negative
Skin biopsy	Subepidermal blister with partial and focal full thickness necrosis of the epidermis, with scattered apoptotic keratinocytes; infiltrate with lympho-histiocytic predominance

Ophthalmological assessment revealed bilateral fluorescein-staining strands of mucus adherent to the anterior surface of the cornea with diffuse conjunctival congestion. He was started on moxifloxacin and prednisolone eye drops.

**Fig. 2:**
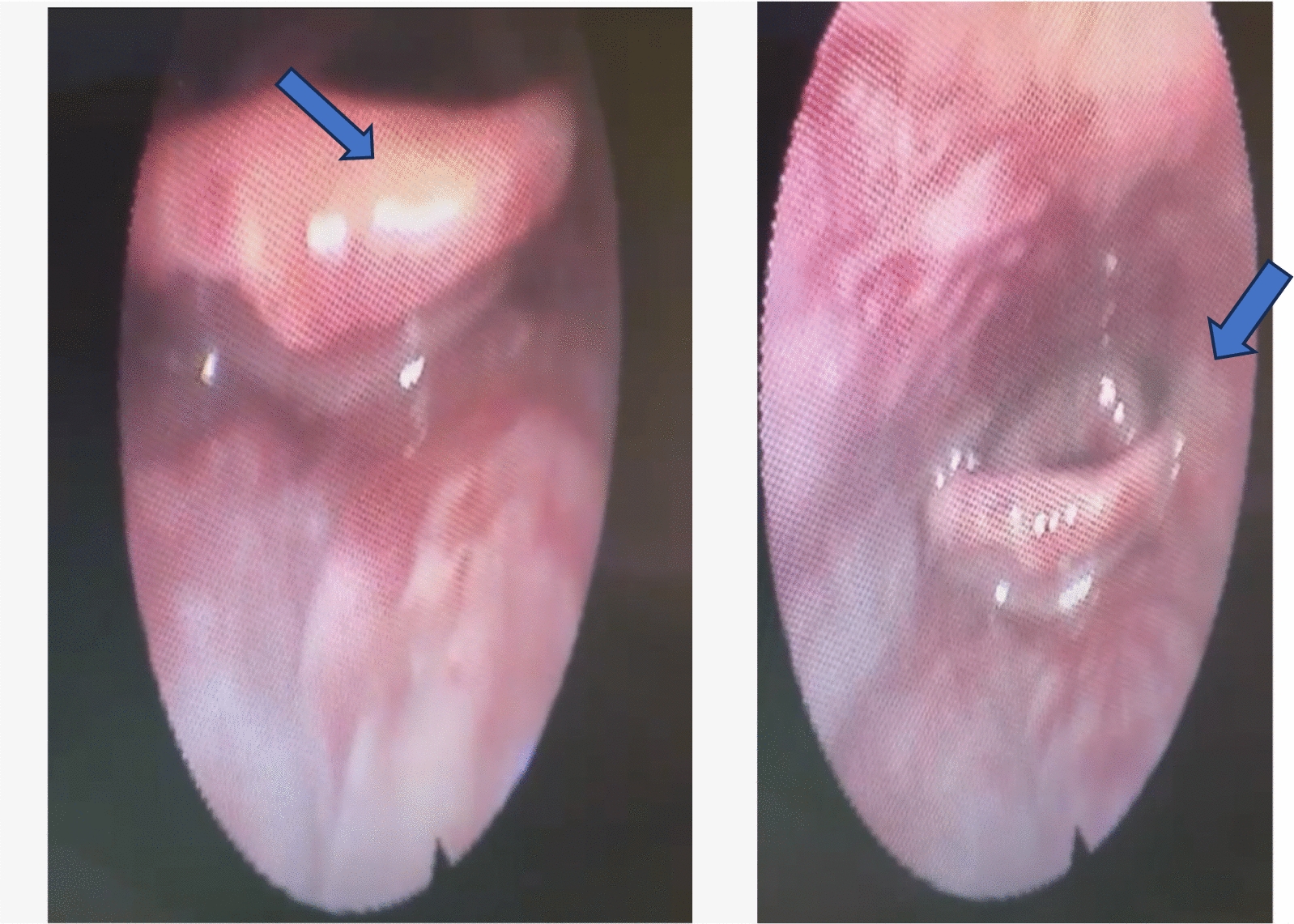
Fiberoptic laryngoscopy showing extensive ulceration involving the vallecula (blue arrow) and posterior pharyngeal wall (blue arrow)

Similarly, otolaryngological evaluation using fiberoptic laryngoscopy (Fig. 2) was performed. Significant findings included superficial ulceration and discharge in the nasopharynx lymphoid tissue, alongside multiple ulcerations over the posterior pharyngeal wall, base of tongue, vallecula, and arytenoid. Management entailed a 5-day course of xylometazoline nasal drops and triamcinolone oral wash.

Subsequent investigations including blood and wound cultures yielded negative results. Anti-skin antibody testing was also negative for pemphigus and pemphigoid (Table 2).

**Fig. 3:**
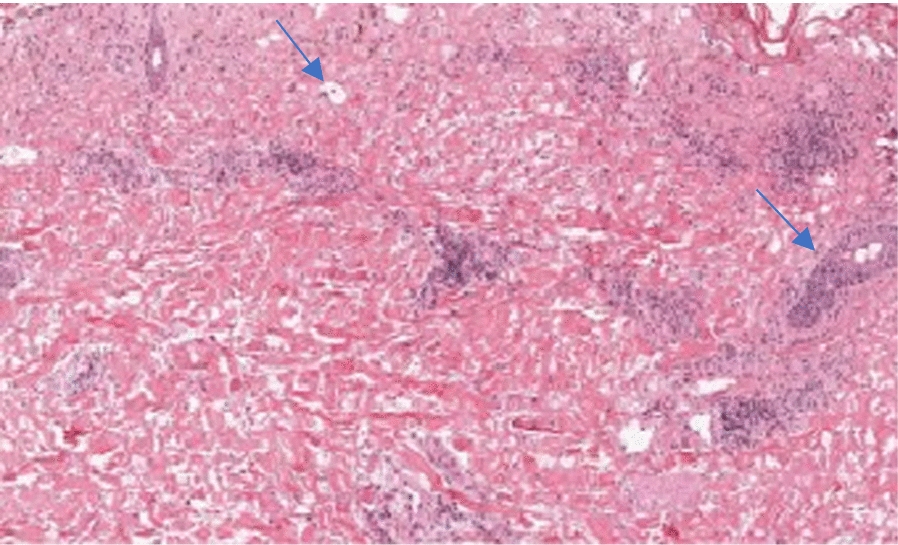
Histopathology of skin: showing Subepidermal blister and partial & focal full-thickness necrosis of the epidermis (thin blue arrows)

Skin punch biopsy showed a subepidermal blister with separation of the epidermis from the dermis with partial and focal full-thickness necrosis of the epidermis, along with scattered apoptotic keratinocytes within the rest of the epidermis (Table 2). The subepidermal blister showed fibrin and mainly lympho-histiocytic infiltrate with few neutrophils and eosinophils (Fig. 3). These findings were consistent with and confirmed the diagnosis of Stevens–Johnson syndrome secondary to Mycoplasma pneumoniae infection.

The patient’s diet gradually advanced from clear fluids to full diet as his odynophagia improved. He received daily oral, ocular, and skin care regimens.

After 13 days of inpatient treatment, he was discharged on a regimen of cyclosporine combined with prednisolone (Table 1), with close follow-up scheduled in the dermatology outpatient clinic.

## Discussion

Mycoplasmas are the smallest self-replicating organisms with the smallest genomes, and even so, the *M. pneumoniae* species is credited to being one of the most common causes of atypical pneumonia [1, 2]. Although the majority of infections are mild, extrapulmonary complications, especially those with mucocutaneous manifestations, have been increasingly reported. *Mycoplasma pneumoniae* is the infectious agent most commonly associated with Stevens–Johnson syndrome [3, 6].

Stevens–Johnson syndrome (SJS) is an acute, rare, and unpredictable immunologic phenomenon characterized by potentially fatal skin reactions, mucous membrane involvement, and associated systemic symptoms. The vast majority of SJS cases have been attributed to be triggered by drugs, while a minority have implicated infectious agents such as *Mycoplasma pneumoniae* and Coxsackievirus A6 as their cause [4].

Most cases of SJS caused by Mycoplasma pneumonia have been described in children and young people with a mean age of 11.9 years and a significant male predominance (66%) [5]. Upon statistical analysis, adults were noted to have developed mucocutaneous manifestations on the same day that the fever and respiratory symptoms commenced, while younger people (< 20 years) had a lag period before the mucocutaneous lesions surfaced [6]. This observation suggests that there had been a prior episode of sensitization to *M. pneumoniae* before the development of SJS upon subsequent exposure.

Mucocutaneous eruptions involving two or more sites and implicating < 10% body surface area has been widely described as the hallmark of SJS induced by *M. pneumoniae* by many authors [7–9]. Cutaneous involvement shows a highly variable presentation with multiple intensities from moderate (19%) or sparse (47%) to absent (34%) [5]. The most common manifestation is that of a rash of macules, purpura, atypical target lesions and flaccid blisters, which start off centrally and then coalesce, spreading to involve the face and extremities [9]. Simultaneous extensive mucosal involvement of two or more sites is a classic finding, with the highest rate of engagement seen with the oral cavity (94%), eyes (82%), and urogenital system (63%) [5].

Another increasingly described dermatological variant seen in conjunction with *M. pneumoniae* is the so-called atypical SJS, which manifests as severe mucositis without any apparent skin lesions. In recent times, this variant has been referred to as *M. pneumoniae*-associated mucositis [10].

A higher number of mucus membranes are affected in *M. pneumoniae*-associated SJS in comparison with drug-induced SJS [10], with ocular lesions being the most commonly incriminated. Adults (> 20 years) demonstrated a much higher affinity for developing ophthalmic sequelae, including synechiae formation [6]. The disease course and manifestations were found to be milder, with less incidence of severe organ involvement affecting the cardiovascular, pulmonary, and hepatic systems in cases of SJS induced by Mycoplasma pneumoniae. This was reflected in the shorter duration of hospital stay and trivial rates of ICU admissions among these patients [10].

The diagnosis of SJS quintessentially depends on a comprehensive history denoting exposure to infections and any recent drug use to delineate any potential causes of adverse drug reaction. Significantly higher levels of erythrocyte sedimentation rate (ESR) were noted with Mycoplasma infection compared with drug-induced cases of SJS [7, 11]. When it comes to diagnostic testing, a combination of polymerase chain reaction (PCR) and enzyme immunoassay (EIA) is the mainstay. EIA measures antibodies to antigen P1 and/or P116 on the *M. pneumoniae* plasma membrane and requires 7–10 days of infection for accurate measurement. Hence, PCR is more useful during the initial stages of an acute infection, as it is more sensitive and specific and detects *M. pneumoniae* DNA rather than antibodies [12].

A skin biopsy could be considered as the gold standard and should be performed to confirm the diagnosis as well as to exclude other possible etiologies. On biopsy, *M. pneumoniae*-induced SJS has been reported to show epidermal necrosis (62%), full-thickness necrosis (46%), subepidermal bullae (77%), and moderate/dense dermal infiltrate (85%) with lymphocytes. These features are also concurrent with the results obtained in our case as well. In contrast, histologic features found in drug-induced cases included individual necrotic keratinocytes and dense dermal infiltrate with a substantial number of eosinophils or neutrophils [10].

The cornerstone of management includes supportive care and adequate fluid replacement with concurrent use of antibiotics, preferably macrolide or doxycycline [7, 13]. Early introduction of antibiotics with regard to the atypical pneumonia reduces the Mycoplasma antigenic stimuli but has no evidence pertaining to its effects on decreasing the incidence of subsequent mucocutaneous involvement [9]. Administration of systemic corticosteroids, intravenous immunoglobulin, and cyclosporine could be considered in severe cases [8, 13].

## Conclusion

Stevens–Johnson Syndrome is an acute and debilitating condition that requires prompt and timely management to ensure minimum morbidity of the patient. The similarities and overlap of features between SJS caused by infectious and drug-related etiologies pose a diagnostic challenge for physicians, which needs to be subdued using systematic research and evaluation with subsequent formulation of an evidence-based assessment and management plan to ensure safe and efficacious medical care for the patients.

## Data Availability

The data that support the findings of this study are available in this article.
